# Epimorphic Regeneration of Elastic Cartilage: Morphological Study into the Role of Cellular Senescence

**DOI:** 10.3390/biology12040565

**Published:** 2023-04-07

**Authors:** Yana Valieva, Aleksandra Igrunkova, Alexey Fayzullin, Natalia Serejnikova, Aleksandr Kurkov, Nafisa Fayzullina, Dina Valishina, Alesia Bakulina, Peter Timashev, Anatoly Shekhter

**Affiliations:** 1 Institute for Regenerative Medicine, Sechenov First Moscow State Medical University (Sechenov University), 8-2 Trubetskaya St., 119991 Moscow, Russia; valieva_ya_m@student.sechenov.ru (Y.V.); fayzullin_a_l@staff.sechenov.ru (A.F.);; 2 World-Class Research Center “Digital Biodesign and Personalized Healthcare”, Sechenov First Moscow State Medical University (Sechenov University), 8-2 Trubetskaya St., 119991 Moscow, Russia; 3 Department of Human Anatomy and Histology, N. V. Sklifosovsky Institute of Clinical Medicine, Sechenov First Moscow State Medical University (Sechenov University), 8-2 Trubetskaya St., 119991 Moscow, Russia

**Keywords:** epimorphic regeneration, elastic cartilage, cellular senescence, lectins, elastin

## Abstract

**Simple Summary:**

The complete regeneration of organs after damage is a distant future of regenerative medicine. One of the few rare mammal models of structural tissue restoration is the defect of the elastic cartilage plate in rabbit ear. Understanding why a complex tissue heals into itself and not into a scar brings us closer to controlling innate regenerative potential. In this experiment, we discovered that the regeneration of the elastic cartilage plate was associated with the presence of cells with the senescence-associated secretory phenotype (SASP). We investigated how the presence of these cells was defined by the defect size, and how they correlated with elastic fiber production and clinical outcome. It is possible that SASP cells regulate the structural reparation of the damaged tissue by paracrine signaling, making them a potential pharmaceutical target.

**Abstract:**

Control over endogenous reparative mechanisms is the future of regenerative medicine. The rabbit ear defect is a rare model which allows the observation of the epimorphic regeneration of elastic cartilage. However, the mechanisms of phenotypical restoration of this highly differentiated tissue have not been studied. We modelled circular ear defects of different sizes (4, 6, and 8 mm in diameter) in 12 laboratory rabbits, and observed them during 30, 60, 90, and 120 day periods. Excised tissues were processed and analyzed by standard histological methods and special histochemical reactions for senescence associated-β-galactosidase and lectin markers. We demonstrated that larger defects caused significant elevation of senescence associated-β-galactosidase in chondrocytes. The fullness of epimorphic regeneration of elastic cartilage depended on the activation of cellular senescence and synthesis of elastic fibers. Further investigation into the role of cells with senescence-associated secretory phenotype in damaged tissues can present new targets for controlled tissue regeneration.

## 1. Introduction

The main goal of regenerative medicine is to restore damaged or lost anatomical structures of the human body by controlling intrinsic regeneration mechanisms and introducing cell technologies, biomaterials, and drugs with pro-regenerative properties [1,2]. However, there is a significant lack of knowledge in the area of endogenous regeneration mechanisms which can be artificially stimulated.

Mammals have specialized types of connective tissues such as the skin dermis, tendon, and hyaline cartilage [3]. Complete functional and structural recover of the highly differentiated tissues is impossible after damage [4]. The defects of these tissues heal by replacement with fibrotic scars (regeneration by substitution) instead of epimorphic (complete) reparative regeneration [5,6,7]. This leads to the development of hypertrophic and keloid scars of the skin, cicatricial changes in tendons, osteoarthrosis, urethral strictures, and tracheal stenosis [8,9,10,11]. Understanding of the molecular and cellular mechanisms that determine the “switch” between programs of epimorphic reparative regeneration and fibrotic substitution presents a challenge for biologists and physicians [12].

Regeneration mechanisms can provide epimorphic restoration of complex organs, for example in some animal species (e.g., salamanders’ limbs, jaws, lens and large sections of the heart) [13,14]. It is believed that mammals, with extremely rare exceptions, are deprived of this ability or have the ability to restore only parts of organs that are insignificant in volume [15]. It is known, however, that African spiny mice are capable of the complete regeneration of hair follicles, sebaceous glands, dermis, and cartilage in ear hole defects [16,17]. Another example is the regeneration of the antlers of deer and moose, which can grow at a rate of over an inch per day [15]. The factors that facilitate epimorphic regeneration in these animals are unknown. In this regard, the discovery of the mechanisms involved in the regulation of epimorphic regeneration in mammals could be possibly translated into regenerative medicine practices.

Cellular senescence is a well-studied mechanism of cell cycle arrest which plays an intrinsic role in tissue regeneration. It is known that levels of cellular senescence markers increase during the regeneration of damaged tissues [18,19,20]. However, it cannot be stated unequivocally that cellular senescence accelerates reparative processes [19,20,21]. Mechanical stress is one of the factors that induces senescence in cells which stop dividing and acquire the senescence-associated secretory phenotype (SASP). In its turn, these cells affect nearby cells through paracrine signaling molecules, such as cytokines and chemokines [22,23]. SASP cells promote clearance of damaged cells by attracting macrophages, and activate the proliferation of neighboring cells [24]. Eventually, the wound is cleaned of senescent and dead cells, and the regeneration process is accelerated [25]. On the other hand, long-term persistence of senescent cells inhibits the proliferative processes in regenerating tissues [26]. It is known that cellular senescence can suppress fibrosis during wound healing [27,28]. Understanding the role of senescent cells can help develop new approaches for the pharmaceutical control of wound healing and foreign body reaction, since fibrosis is based on a complex of cell transdifferentiations as well [29,30]. The regeneration outcome is dependent on cellular senescence, which is activated by mechanical stress defined by the defect [31]. We expect that cellular senescence induced by mechanical factors (for example, excessive or insufficient mechanical stress) can become a pharmaceutical target providing control over the course of the reparative processes in patients [9,31].

Models of organ-specific regeneration have seen increasing attention in recent years [31,32]. In this project, we used one of the few available experimental models of epimorphic regeneration in mammals, namely, a model of full-thickness wounds on rabbit ears [33,34,35]. This model involves the formation of holes in ears with biopsy punch tools, in combination with the optional preservation of the surrounding perichondria and skin. It is known that the rabbit ear cartilage plate can fully regenerate under certain conditions [33]. The size, type (volume), and location of the defect play significant roles in the healing of full-thickness defects in auricular cartilage in rabbits [32,33]. The outcome of the wound healing can be either the complete restoration of the tissues of the ear, or the partial replacement of them with a scar. The factors controlling the regeneration of rabbit ear cartilage are understudied [34,36].

The key hypothesis of this project is that the rate and outcome of regeneration depend on the mechanical stress (size of the defect) through cellular senescence of chondrocytes in damaged tissues of rabbit ear cartilage plate. In this study, we want to establish how and to what extent the epimorphic and substitutional reparative regeneration of the ear cartilage is controlled by the mechanisms of cellular senescence. Investigation into the mechanisms of the epimorphic regeneration of elastic cartilage will allow us to identify fundamentally new targets for the directed regeneration of complex differentiated tissues.

## 2. Materials and Methods

### 2.1. Surgical Technique

The experiment in twelve chinchilla rabbits (males, 2–2.5 kg) was approved by the Local Ethical Committee of Sechenov University (Protocol #13-22/22.06.2022). The rabbits were kept under the standard vivarium conditions, one animal per cage, and were provided with complex granulated laboratory chow and constant access to water.

For the surgery, the animals were anaesthetized by intramuscular injection of a solution of Zoletil 100 (Vibrac, France; 6 mg/per 1 kg of animal body weight), supplemented with local anesthesia of the operating field with a solution of Novocain 0.5%. Full-thickness defects were created using 4, 6, and 8 mm biopsy punchers (Dermo Punch, Sterylab, Milan, Italy). Three punch holes of different diameters were modelled on each rabbit ear at a distance of 15 mm from the marginal artery and each other. The wounds were dressed with Cosmopor E patches (Paul Hartmann, Heidenheim an der Brenz, Germany) and treated with a 3% solution of hydrogen peroxide for three days.

On the 30th, 60th, 90th, and 120th postoperative days (POD30, POD60, POD90, and POD120, respectively) the rabbits were euthanized by the injection of a solution of Zoletil 100 (Vibrac, Carros, France; 60 mg/kg of animal body weight).

The sites of the defects were dissected together with the surrounding tissues at approximately 2–3 mm from the original wounds’ edges. Each of the dissected samples was divided into two parts: a half of each sample was fixed in 10% neutral buffered formalin; another half of the original sample was immersed in an O.C.T. cryogel (Fisher Healthcare, Pittsburgh, PA, USA) and snap-frozen in liquid nitrogen for cryobanking.

### 2.2. Macroscopic Assessment

Gross examination of the defects was conducted during the first two weeks of the experiment and also before the resections on POD30, POD60, POD90, and POD120. Wound healing features were described by the following score system (Table 1). The sums of macroscopic scores of each defect were used for statistical analysis.

### 2.3. Histological Processing and Analysis

Four-μm-thick sections of the formalin-fixed-paraffin-embedded tissue samples were stained with hematoxylin and eosin (H and E); orcein for the detection of elastic fibers; and with Picrosirius red (PSR) for the detection of collagen fibers. A LEICA DM4000 B LED microscope, equipped with a LEICA DFC7000 T digital camera running under the LAS V4.8 software (Leica Microsystems, Wetzlar, Germany) was used for the examination and imaging of the samples. The specimens were studied by methods of standard (for H and E, orcein, and PSR stained samples) and polarized light (PSR stained samples) microscopies. Morphometric analysis of the histological samples (72 sections for each staining protocol) was performed by two blinded pathologists. Discrepancies in their results were resolved by a third pathologist. This pathologist had knowledge of the samples’ group belonging and wrote the histological report for each study group.

To evaluate the total quality of epimorphic regeneration, we used the semi-quantitative score system on POD 120 (Table 2).

Distances between the edges of regenerating fibrous cartilage were measured for the evaluation of the defect size. Areas of regenerating cartilage tissue at both edges of the cartilage plate were measured for further evaluation. The ratio of the regenerates calculated as a relation of the smaller value of the edge area of the regenerating cartilage to the larger one was used to study the uneven distribution of the regeneration tissue. We used basic measuring tools of LAS V4.8 software (Leica Microsystems, Wetzlar, Germany) for these purposes.

The content of elastic fibers in the regenerating cartilage was evaluated in each sample (Table 3). We analyzed elastic fiber regeneration by summarizing the scores from both defect edges.

### 2.4. Analysis of Senescence Associated-β-Galactosidase (SA-β-Galactosidase) Expression

Ten-µm-thick sections were obtained on a cryotome, fixed with 4% formalin solution, washed four times with 10 mM citrate buffer (pH = 6.0), and stained with a pre-prepared solution consisting of citrate buffer (pH = 6.0)), NBT (0.4 mg/mL, D298839.00005, Dia-M, Moscow, Russia) diluted in 70% DMFA (227056, Sigma-Aldrich, Waltham, MA, USA), and X-gal powder (1.0 mg/mL, R0404, Fermentas, Waltham, MA, USA) dissolved in 100% DMFA (227056, Sigma-Aldrich, USA).

SA-β-galactosidase expression was evaluated by intensity and number of positively stained cells in regenerating cartilage tissue via the following system (Table 4). The final expression score was estimated as a sum of intensity score and cell quantity score.

### 2.5. Histochemical Lectin Reaction

We studied glicoprotein profiles of tissues with a lectin kit that included RCA_120_, WGA, UEA I, and SBA (Lectin Kit I, Biotinylated, BK-1000, Vector Laboratories, Mowry Ave Newark, CA, USA).

Four-µm-thick sections were prepared and underwent a standard deparaffinization, dehydration, and rehydration procedure followed by heat-induced epitope retrieval (pH = 6.0 sodium citrate solution for 30 min in a water bath at 70 °C). Then 3% H_2_O_2_ was added to deactivate endogenous peroxidase. Avidin-biotin blocking complex (SP-2002, Vector Laboratories, USA) was used to prevent the binding of lectins to endogenous biotin. A carbo-free blocking solution (SP-5040, Vector Laboratories, USA) was used to prevent non-specific protein interaction. The sections were stained with biotinylated lectins (50 µg/mL). Tissue sections were treated with a solution of streptavidin conjugated with horseradish peroxidase (Vectastain Elite ABC Kit, Peroxidase Standard, PK-6100, Vector Laboratories, USA). Sections were washed with PBS with TWEEN 20 (P1379-1L, Sigma-Aldrich, USA), treated with diaminobenzidine (34002, Thermo Scientific, Waltham, MA, USA), and counterstained with hematoxylin.

We evaluated RCA_120_ staining by the following score system (Table 5).

### 2.6. Statistical Analysis

The statistical analysis of the experimental quantitative data was performed with a standard program package, GraphPad Prism version 8.00 for Windows (GraphPad Software, Inc., San Diego, CA, USA). The normal distribution of the quantitative data (regenerated defect length, intermarginal relation of regenerated areas, area of regenerated cartilage) was checked by Shapiro–Wilk’s normality test. The intergroup differences in the quantitative data (regenerated defect length, intermarginal relation of regenerated areas, area of regenerated cartilage) were analyzed by the two-way ANOVA method, followed by Tukey’s multiple comparison test. The search for the differences in the histological scores (macroscopic evaluation of regeneration, epimorphic regeneration of elastic cartilage, SA-β-galactosidase expression, elastic fiber content, RCA_120_ staining) was conducted using the Kruskal–Wallis test, followed by Dunn’s multiple comparison test. The statistical analysis results were presented as column graphs of the mean values and standard deviations (SD) for quantitative data, and as median values and interquartile range for histological score parameters. *p*-values equal or less than 0.05 were considered statistically significant.

## 3. Results

### 3.1. Gross Assessment of Wound Healing

**Figure 1:**
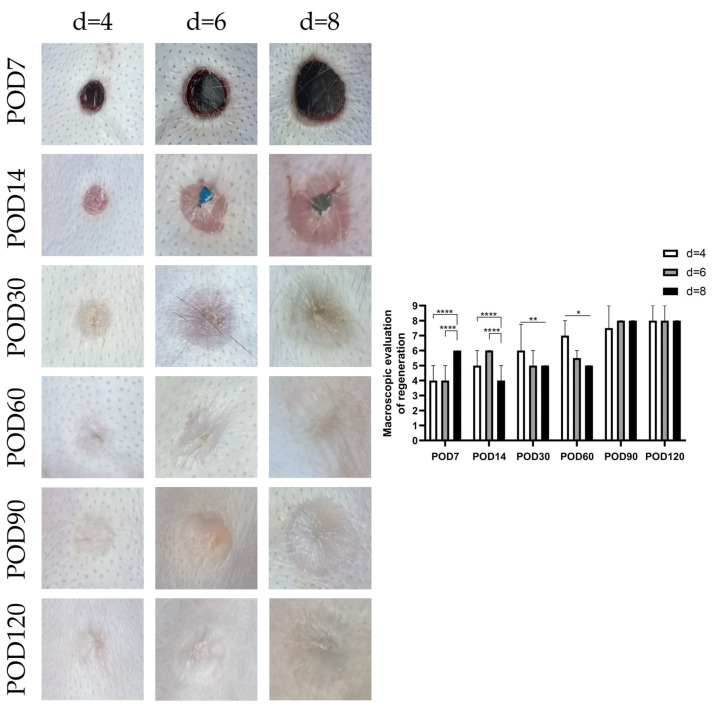
Gross assessment of healing defects over the 4 month period of the experiment. Statistical analysis of macroscopic evaluation of regeneration, median values ± interquartile range, *—*p* ≤ 0.05, **—*p* ≤ 0.01, ****—*p* ≤ 0.0001.

On the 1st day, a minor volume of odorless and transparent exudate was observed in all defects. The wound edges were even and smooth. On the 2nd and 3rd days, the edges of the defects obtained a red color. On the 7th day, the defects had even contours and thickened edges bulging above the skin surface (Figure 1).

On the 14th day, 4 mm diameter defects (d = 4) visually completely healed: wounds were consolidated and newly formed tissue was red and dense. In 6 mm and 8 mm diameter defect (d = 6 and d = 8) groups, wounds were not consolidated. The edges of the wounds were epithelialized, and accumulated horny masses.

On the 30th day, all defects were completely epithelialized and visually consolidated. In the d = 4 group, the newly formed tissue had an elastic consistency and a smooth, pale surface. In the d = 6 and d = 8 groups, the defects had a pigmented surface and retractions in the central part, which deformed the surrounding tissues. On the 60th day of the experiment, the dynamics of wound healing were not significant in all groups.

A complete normalization of wound color was observed in all study groups 3 months after the modelling of the defects. However, the tissue replacing the defects was characterized by an extremely dense texture resembling bone in half of the cases in the d = 4 and d = 8 groups. In the d = 6 group, the newly formed tissue had the same density as the surrounding tissues. Wounds did not change significantly on the 120th day of the experiment.

### 3.2. Histological Analysis

**Figure 2:**
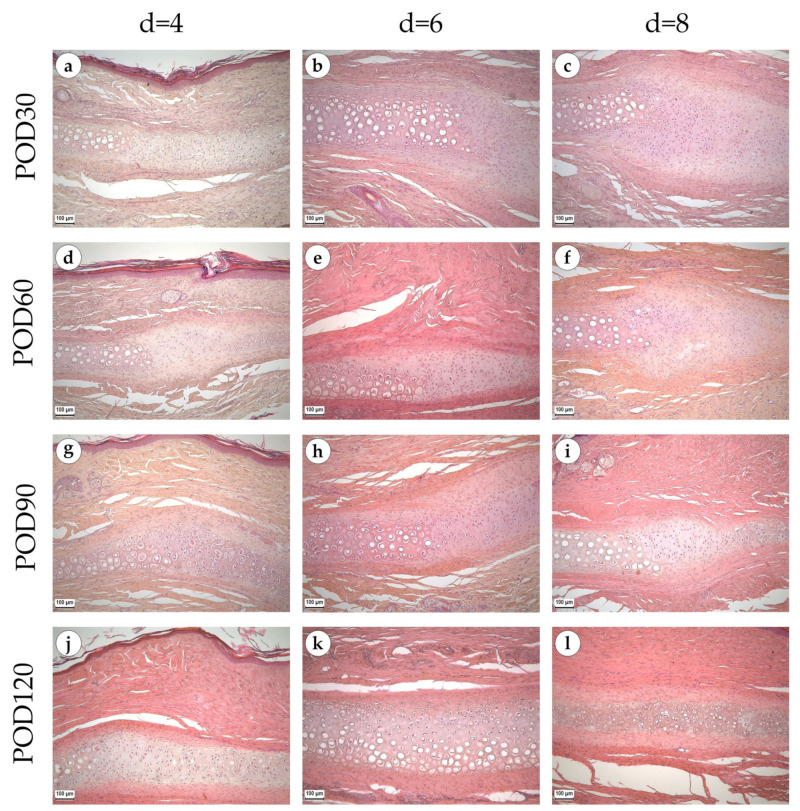
Histological examination of regenerating wound defects over 120-day period of the experiment, H and E staining, scale bar—100 µm, bright field microscopy. Columns depict the studied groups (d = 4, d = 6, and d = 8 wounds).

On POD30, the wound surface was lined with a continuous keratinized stratified epithelium in all samples in the d = 4 and d = 6 groups (Figure 2a,b). In the d = 8 group, the wound edges were separated by a thin epithelialized fissure in half of the samples (Figure 2c). Dense immature fibrotic tissue filled the space between the edges of the regenerating cartilage plate. It consisted of fibroblasts and blood vessels surrounded by collagen fibers oriented parallel to the skin surface. Peripheral parts of the subepithelial derma located above the regenerating cartilage tissue contained skin appendages surrounded by reticular dermis, and resembled an intact skin. The defects had minimal or no immune cell infiltration areas, indicating the absence of local infection.

**Figure 3:**
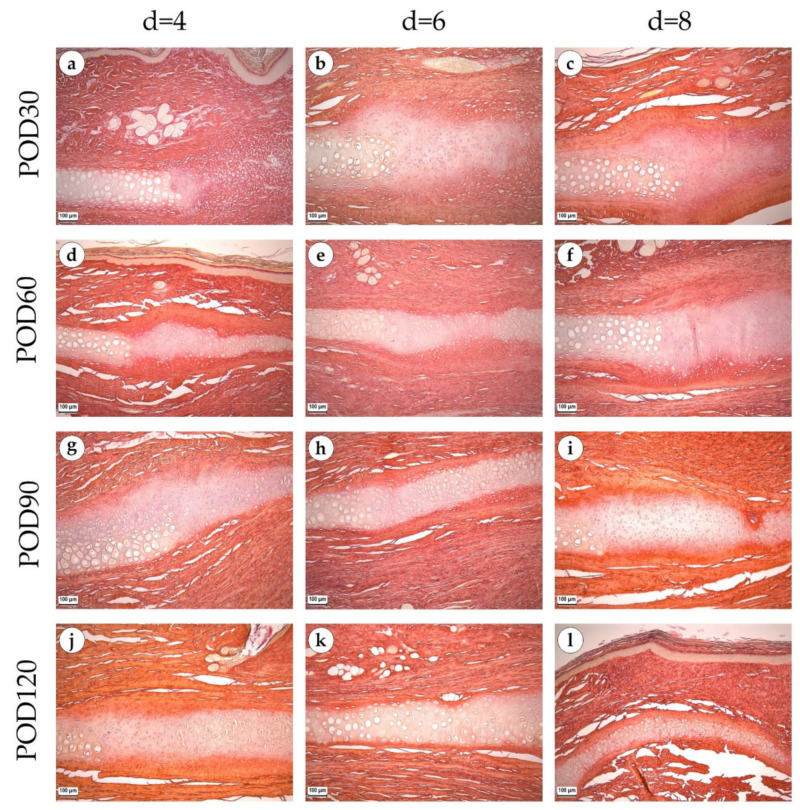
Histological examination of collagen content in regenerating wound defects over 120-day period of the experiment, PSR staining, scale bar—100 µm, bright field microscopy. Columns depict the studied groups (d = 4, d = 6, and d = 8 wounds).

**Figure 4:**
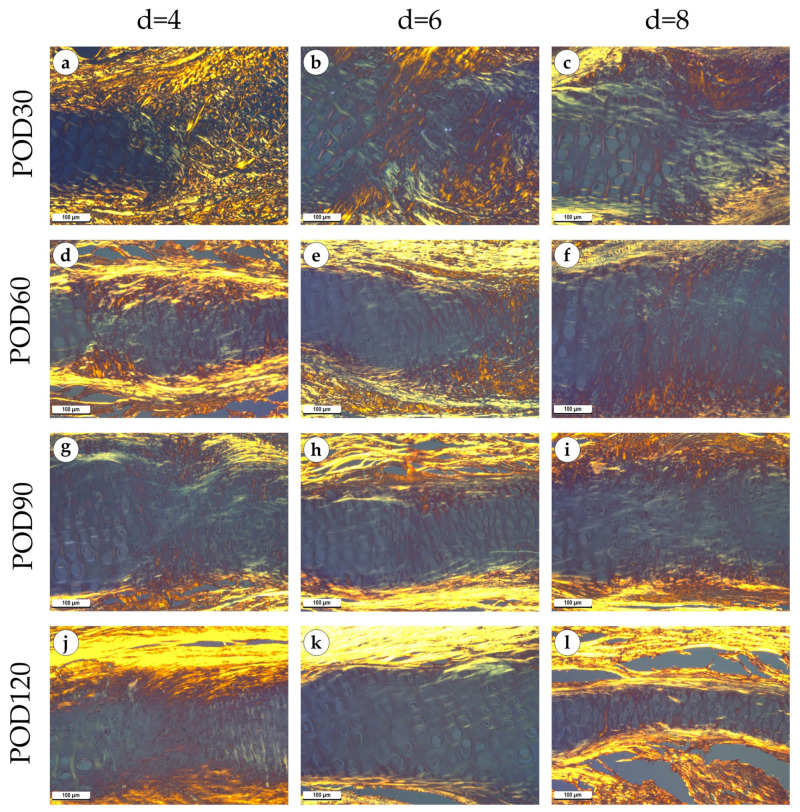
Histological examination of anisotropic characteristics of collagen in regenerating wound defects over 120-day period of the experiment, PSR staining, scale bar—100 µm, polarized light microscopy. Columns depict the studied groups (d = 4, d = 6, and d = 8 wounds).

**Figure 5:**
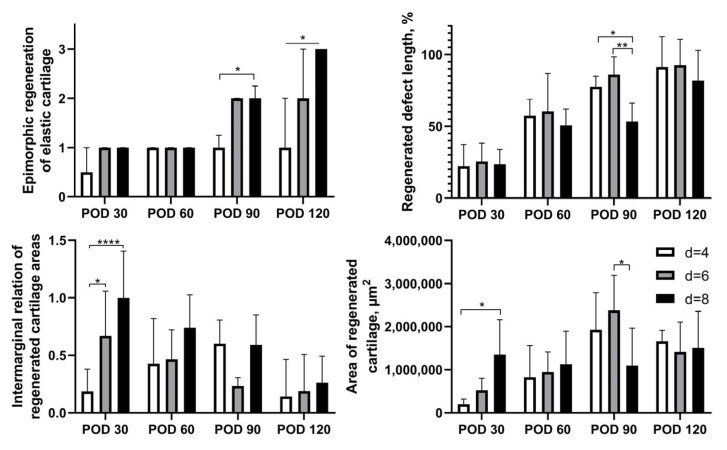
Statistical analysis of morphological findings. Epimorphic regeneration of elastic cartilage, median values ± interquartile range. Regenerated defect length, mean values ± SD. Intermarginal relation of regenerated cartilage areas, mean values ± SD. Area of regenerated cartilage, mean values ± SD, *—*p* ≤ 0.05, **—*p* ≤ 0.01, ****—*p* ≤ 0.0001.

The cartilage plate regenerated by forming thick fibrous cartilage extensions on its edges. These extensions consisted of polygonal and round chondrocytes surrounded by collagen fibers. The areas of regenerating cartilage were larger on one edge of the plate than the other in the d = 4 and d = 6 groups. Fibrous cartilage tissue formed a prolonged layer located between regenerating skin tissues of the ventral and dorsal surfaces of the ear. Regenerating cartilage tissue growing from resected edges in animals of the d = 6 and d = 8 groups was significantly thickened due to appositional growth and proliferation of the cells of the inner layer of the perichondrium. In these groups, areas of regeneration were less positively stained with Picrosirius Red than in the d = 4 group. This finding indicated a lower collagen content in them, resembling intact elastic cartilage (Figure 3a–c). The fibrous cartilage contained numerous anisotropic fibers surrounding chondrocytes visualized as red and yellow fibers under polarized light (Figure 4a–c). The areas of low Picrosirius Red staining corresponded with mild green anisotropy under polarized light, indicating the absence of thick fibers (Figure 5).

**Figure 6:**
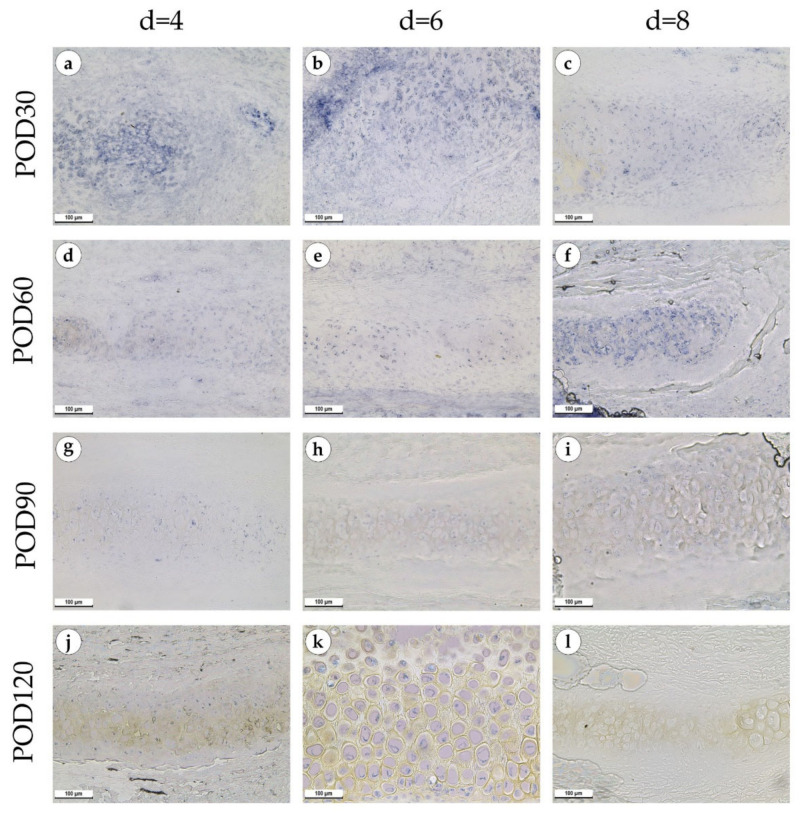
Histochemical detection of SA-β-galactosidase in regenerating wound defects over 120-day period of the experiment, X-gal staining, pH = 6.0, scale bar—100 µm, simple light microscopy. Columns depict the studied groups (d = 4, d = 6, and d = 8 wounds).

**Figure 7:**
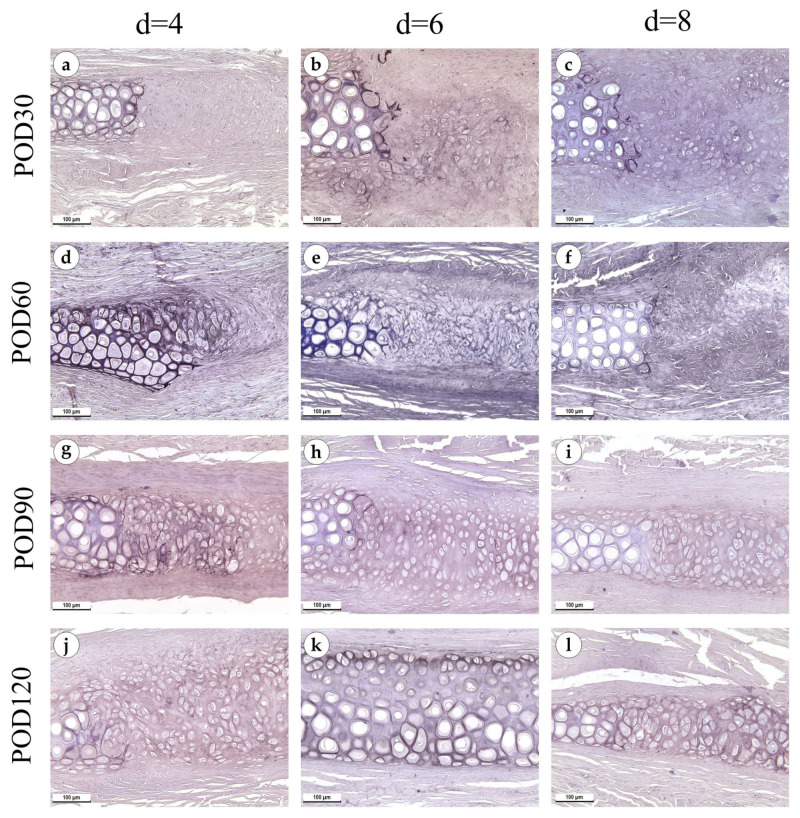
Histological examination of elastic fiber content in regenerating wound defects over 120-day period of the experiment, orcein staining, scale bar—100 µm, simple light microscopy. Columns depict the studied groups (d = 4, d = 6, and d = 8 wounds).

**Figure 8:**
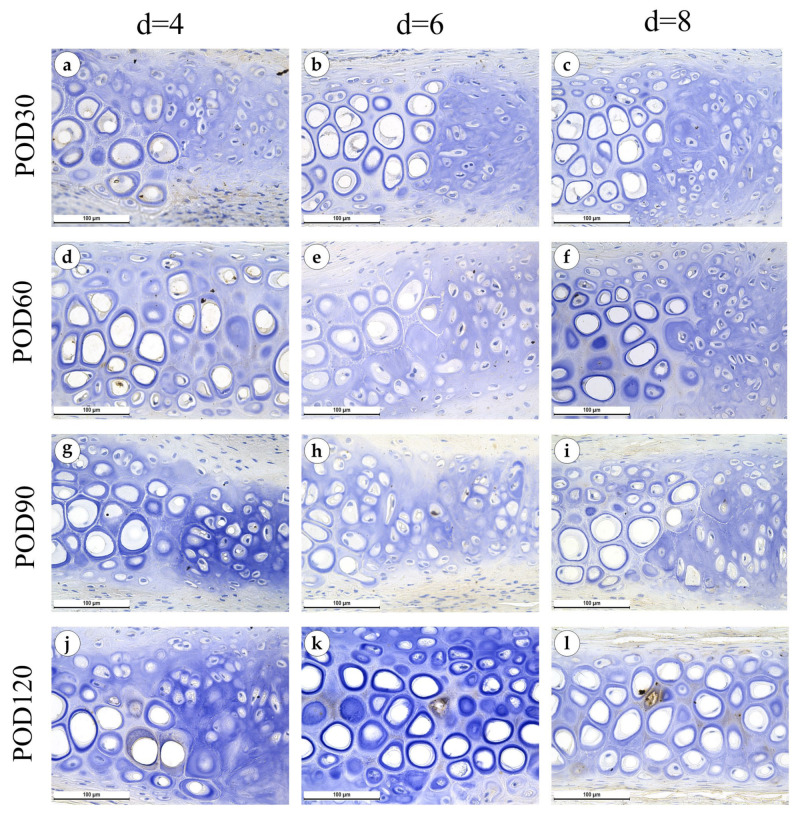
RCA_120_ binding patterns in regenerating wound defects over 120-day period of the experiment, RCA_120_ lectin staining, scale bar—100 µm, simple light microscopy. Columns depict the studied groups (d = 4, d = 6, and d = 8 wounds).

**Figure 9:**
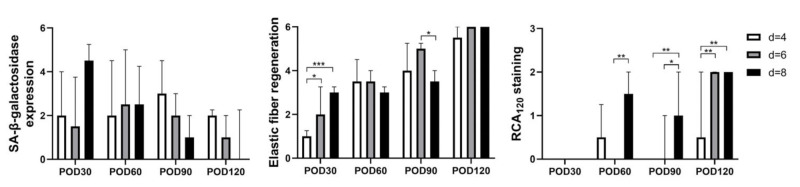
Statistical analysis of SA-β-galactosidase, elastic fibers and RCA_120_ lectin staining levels, median values ± interquartile range, *—*p* ≤ 0.05, **—*p* ≤ 0.01, ***—*p* ≤ 0.001.

**Figure A1:**
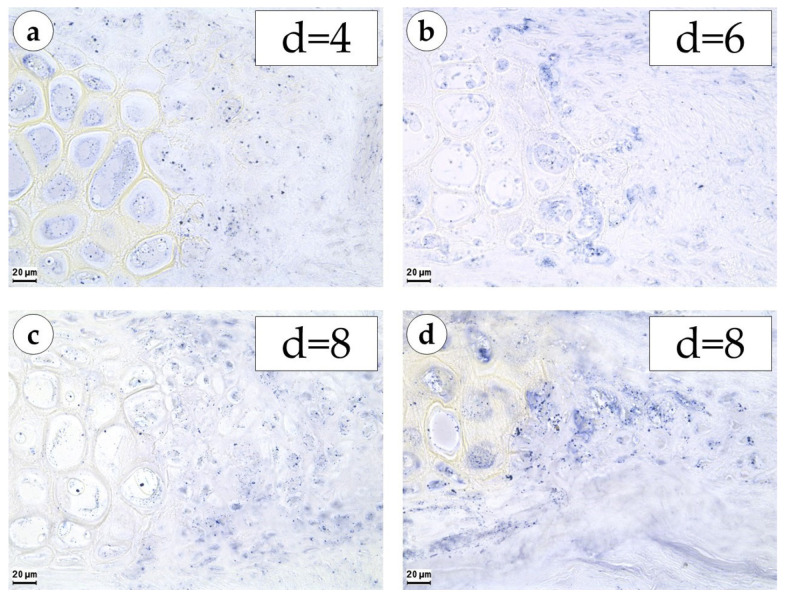
Histochemical detection of SA-β-galactosidase in regenerating wound defects of 4 mm (**a**), 6 mm (**b**) and 8 mm (**c**,**d**) sizes on POD30, X-gal staining, pH = 6.0, scale bar—20 µm, simple light microscopy.

Moderate SA-β-galactosidase expression was observed in groups of cells of the regenerating elastic cartilage in the d = 4 and d = 6 groups (Figure 6a,b and Appendix A Figure A1). The expression was localized in the extracellular matrix of fibrous cartilage as well. In contrast, expression of SA-β-galactosidase in the d = 8 group was detected in a majority of cells in all areas of newly formed fibrous cartilage (Figure 6c). Cartilage regenerate lacked elastic fibers and was almost completely represented by fibrous cartilage in the d = 4 group (Figure 7a). In some samples, small islands of lacunae surrounded by elastic fibers were noted. In the d = 6 group, small areas of regenerating cartilage located close to the edge of the intact cartilage plate contained elastic fibers (Figure 7b). In the d = 8 group, the formation of foci or layers of cartilage tissue containing elastic fibers was noted in all samples (Figure 7c). RCA_120_ expression was found in the extracellular matrix of intact cartilage, but not inside the regenerating tissue in all experimental groups (median RCA_120_ score = 0) (Figure 8a–c, Figure 9).

On POD60, all defects were consolidated, epithelialized, and they contained hair follicles and skin glands (Figure 2d–f). Granulation tissue reduced in volume and cellular density. It formed reticular dermis under the majority of epithelium lining in the d = 6 and d = 8 groups. Thick bundles of collagen fibers filled the space between regenerating edges. Bone tissue formation was observed in the area of regeneration in two defects in the d = 8 group. Regenerating chondrocytes were predominantly round and formed lacunae in proximity to the intact cartilage plate. Focuses of regenerating cartilage with weak Picrosirius Red staining were observed more often than on POD30 (Figure 3d–f). However, only a thin central part of the regeneration layer was weakly stained in the d = 4 and d = 6 groups. Weakly stained areas were predominant and formed prolonged layers in the d = 8 group. The regenerating cartilage was mostly isotropic in contrast to the surrounding perichondrium and dermis (Figure 4d–f). Red and yellow anisotropic collagen interlayers oriented perpendicularly to the cartilage plate were observed in the central parts of the defects.

The expression of SA-β-galactosidase was significantly lower than on POD30 in the immature elastic cartilage in all study groups. SA-β-galactosidase was detected in singular groups of cells with a minor number of positively stained granules (Figure 6d–f). Areas of concentrated SA-β-galactosidase positive cells were found relatively more often in the d = 8 than in the d = 4 or d = 6 groups, but the difference was not significant. In the d = 4 and d = 6 cartilage regenerates, prolonged layers of elastic cartilage were formed, which was confirmed by the positive staining of the extracellular matrix with orcein (Figure 7d,e). In the d = 8 group, the areas of regenerates with positive orcein staining were vast, but they did not form unilaterally directed layers in most samples (Figure 7f). RCA_120_ expression was detected in singular, sparsely-located chondrocytes near the defect margin in the d = 4 group. One to two RCA_120_ positive cells were found in each margin section (median RCA_120_ score = 0.5) (Figure 8d). No RCA_120_ positive cells were found in the d = 6 group (median RCA_120_ score = 0) (Figure 8e). RCA_120_ positive cells were found in the area of intact cartilage near the defect margin and in chondroblasts of the inner layer of the perichondrium in all cases in the d = 8 group; median RCA_120_ score = 1.5 (Figure 8f).

The regeneration of cartilage plate significantly accelerated after POD 60, resulting in a major growth of fibrous cartilage on POD90 (Figure 2g–i). Chondrocytes became large and more distant from each other in the d = 6 and d = 8 groups. Collagen fibers in the surrounding connective tissue and skin derma became densely packed. The regenerating cartilage was weakly stained with Picrosirius Red and gave green or no anisotropy under polarized light in all groups (Figure 3g–i and Figure 4g–i). Small focuses of bone were observed in two d = 8 defects.

Only singular, sparsely-located chondrocytes and chondroblasts of the inner layer of the perichondrium expressed SA-β-galactosidase. The marker was observed as singular blue granules in a cell. This pattern of expression was observed across the whole volume of newly regenerated cartilaginous tissue (Figure 6g–i). The tinctorial properties of the orcein stained regenerates resembled the intact cartilage in the d = 4 and d = 6 groups (Figure 7g,h). The patterns of the chondron structures were clearly defined, the elastic fibers evenly surrounded the cartilaginous lacunae. In the d = 8 group, foci of orcein-positive regenerating elastic cartilage tissue were formed (Figure 7i). No RCA_120_ positive cells were found in d = 4 (median RCA_120_ score = 0) (Figure 8g). Singular RCA_120_ positive cells were found only in two cases in d = 6 (median RCA_120_ score = 0) (Figure 8h). In intact cartilages in d = 8, one to two RCA_120_ positive cells were observed in each ×400 field of view (median RCA_120_ score = 1) (Figure 8i).

On POD 120, highly anisotropic reticular dermis was observed under the epithelium in all groups (Figure 4j–l). However, the bundles of collagen fibers were less densely packed in d = 6 groups, indicating the slowdown of regeneration. Areas of bone formation were observed inside regenerating cartilage plates in three d = 4 and three d = 8 cases (Figure 2j–l). Maturing elastic cartilage was weakly stained with Picrosirius Red in all groups (Figure 3j–l). Separate foci of complete regeneration were observed in the d = 4 defect, while most of the lacunae were small and polymorphic. The chondron structure of the regenerated cartilage resembled normal elastic cartilage in the d = 6 and d = 8 groups. However, only the d = 6 cartilage plate regenerated on the whole length of the defect.

**Figure 10:**
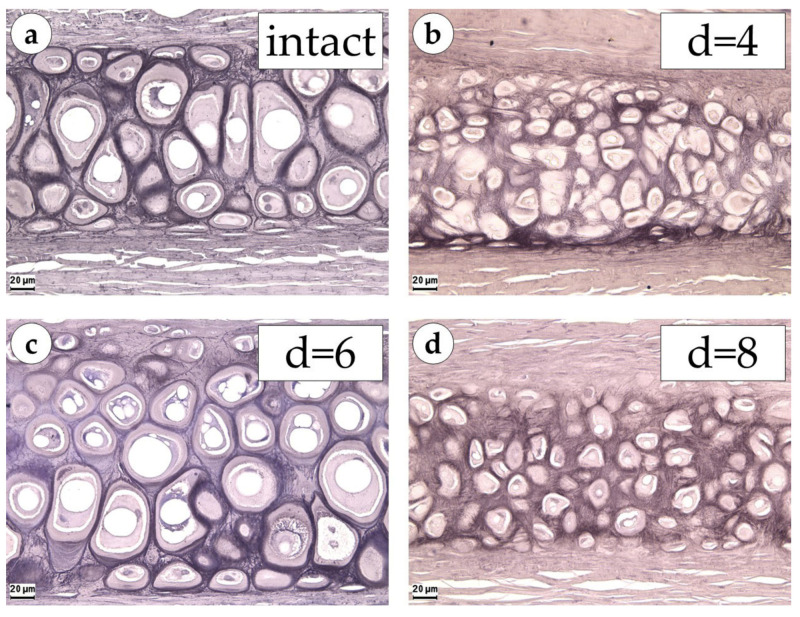
Histological examination of elastic fiber content in regenerating wound defects in proximity to the intact cartilage plate 120 days after the surgery, orcein staining, scale bar—20 µm, simple light microscopy. (**a**) Cartilage plate was covered with perichondrium. The outer region of the elastic cartilage consisted of singular small flattened chondrocytes in lacunae surrounded by bundles of elastic fibers. Large lacunae of the central zone were organized into pairs which were divided by a fine border of intertwining elastic fibers. (**b**) The chondroblasts of inner perichondrium layer were surrounded with aggregations of elastic fibers. The cell density was higher than in the intact cartilage. Chondrocytes were small, had polygonal and oval shapes, and formed isogenic groups. The cells actively synthetized elastic fibers which were observed inside and outside the lacunae. (**c**) Chondrocytes of the outer region were proliferating, and formed numerous isogenic groups. The bulk of the elastic cartilage was made of large lacunae separated by thin bundles of elastic fibers. The regenerated cartilage resembled the intact tissue. (**d**) The perichondrium was thickened due to the growth of connective tissue. The cartilage consisted of singular polygonal chondrocytes in small lacunae. Extracellular matrix occupied larger portion of the cartilage than in other groups. It consisted of elastic fibers forming a dense network of thin, branching elastic fibers.

SA-β-galactosidase was expressed predominantly in the chondroblasts of the perichondrium (Figure 6j–l). Positively stained cells in regenerated elastic cartilage had submembranous blue staining that differed from the granular staining that was evident at earlier time points. This submembranous SA-β-galactosidase expression resembled intact areas of elastic cartilage. In the d = 4 group, areas adjacent to the intact cartilage completely restored the structure of the elastic cartilage, which was confirmed by orcein staining (Figure 10). The edges of the regenerating cartilage showed that regeneration is accompanied by the formation of a mixed fibrous and elastic defectsof cartilage (Figure 7j). In the d = 6 group, complete restoration of the cartilage plate was achieved with restoration of the elastic phenotype of the cartilage. This was confirmed by orcein staining (Figure 7k). In the d = 8 group, there was a preferential restoration of the elastic cartilage plate with the characteristic size and shape of lacunae and the extracellular matrix positively stained with orcein (Figure 7l). RCA_120_ positive cells were observed in regenerating cartilages in half of the cases (median RCA_120_ score = 0.5) (Figure 8j). One to four RCA_120_ positive cells were found in each regenerated cartilage plate in whole sections in d = 6 (median RCA_120_ score = 2) (Figure 8k). Similar to the d = 6 group, regenerated layers of elastic cartilage in d = 8 had numerous RCA_120_ positive cells (median RCA_120_ score = 2) (Figure 8l).

## 4. Discussion

In the present work, we studied the epimorphic regeneration of elastic cartilage using one of the few affordable experimental models, the rabbit ear model of the full-thickness wound [33]. The choice of this model was based on the ability of rabbit auricle defects to heal with full restoration of tissue structures [37]. The restoration of skin, skin appendages, the perichondrium, and cartilage plate underwent the formation of cartilage blastema-cluster of pluripotent cells on the wound surface [17]. We used and adapted this model to find out the key regulating patterns and mechanisms defining one of two possible outcomes, epimorphic and fibrotic reparative regeneration.

In discussing the findings of this study, it is crucial to acknowledge the limitations associated with the model. These limitations include the presence of multiple defects on each ear and the potential dependence of regeneration on defect position. Firstly, the model involves multiple defects on each ear, which may influence tissue reactions due to the complex tissue microstructure. Although we have observed independent tissue reactions when defects were separated from the marginal artery and from each other by at least 15 mm, the presence of multiple wounds could still potentially impact mechanical stress in regenerating tissues. To address this concern, we co-localized wounds of different diameters on the same ear. However, it is worth noting that the possible effects of multiple wounds on tissue regeneration cannot be entirely ruled out. Secondly, our model is limited by the potential dependence of regeneration on the choice of defect position. Although rabbits are considered the next best model animal for studying fibrotic processes in soft tissues (following pigs and sheep), our study assumes that the surgical techniques developed for modelling scar formation can be applied to cartilage plate regeneration. While this assumption forms the basis of our experimental design, it is important to be cautious when extrapolating findings from one tissue type to another, as the underlying biological processes may not be identical.

**Figure 11:**
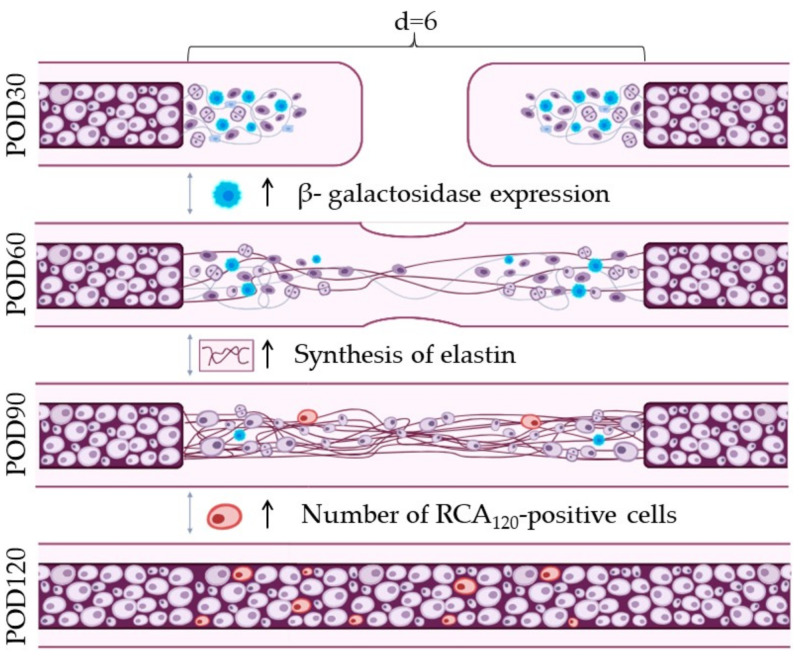
The regeneration of 6 mm diameter defect of cartilage plate on POD30, POD60, POD90, POD120. POD30: mechanical stress induces cellular senescence, cells acquire SASP and accelerate the proliferation of neighboring cells (senescence cells are blue). Newly formed cartilage contains a bulk of collagen fibers. POD60: the number of senescent and damaged cells is reduced due to the clearance by macrophages attracted by SASP substances. Elastic fibers are formed. POD90: the number of senescent cells reduces, the amount of elastic fiber increases, elastic cartilage begins to mature. RCA_120_—positive cells (red cells) appear. Newly formed cartilage is differentiated into elastic type. POD120: An entire layer of elastic cartilage is formed, and the number of RCA_120_ positive cells in the newly formed cartilage increases. POD—postoperative day, SASP—senescence-associated secretory phenotype, RCA_120_—ricinus commuhis agglutinin 120.

We modeled full-thickness rabbit ear wounds of different diameters to evaluate the influence of wound size on rate and type of elastic cartilage regeneration. The results of our experiment demonstrated that epimorphic regeneration of rabbit elastic cartilage depends on the defect size and morphological features of regeneration on POD 30. The defect size directly correlated with the level of expression of SA-β-galactosidase in newly formed cartilage. It is known that mechanical stress makes some cells obtain the senescence-associated secretory phenotype in regenerating cartilage [9]. Such cells are no longer able to divide, but in turn they activate neighboring cells’ proliferation and promote the elimination of damaged cells by the attraction of macrophages and lymphocytes into the area of inflammation [24]. Based on this data, we assumed that the phenomenon of cell senescence induced by mechanical stress is involved in the regeneration of rabbit elastic cartilage according to the senescence–clearance–regeneration model [24,25]. This model can explain why cartilage regeneration was the most intensive in the group with the largest diameter of the defect until POD 60. After that, the level of SA-β-galactosidase decreased in newly formed cartilage. On POD 90, we observed acceleration of cartilage growth in the d = 4 and d = 6 groups, and a significant decrease in the level of SA-β-galactosidase in all groups. Therefore, a reduction in the number of senescent cells was indirectly related to the clearance of damaged cells from the injury site, which prevented regeneration (Figure 11).

At the end of the fourth month, we made a microscopic evaluation of the newly formed cartilage and found that its structure was different depending on defect diameter. In the group with the least diameter, fibrous cartilage prevailed, while there were only singular lacunae of elastic cartilage with small round chondrocytes. The bone tissue was formed in half of the cases. In the d = 6 group, numerous large lacunae were surrounded by elastic fibers, and dystrophic changes were completely absent. The new cartilage tissue with elastic fibers was formed in the group with the largest diameter. Its microstructure resembled the intact cartilage plate. However, regeneration in this group occurred with bone formation and long areas of unrestored cartilage tissue. Apparently, the mechanical stress in the d = 8 defect area was sufficient, but it exceeded the potential of tissue regeneration. Based on our results, we consider that a defect diameter of 6 mm is favorable for the epimorphic regeneration of elastic cartilage in rabbit ear. The morphological and histochemical characteristics of the tissue were as close as possible to those of the normal elastic cartilage tissue. This is most likely due to the optimal rate of regeneration. It leaves the question why the mechanical stress in the d = 4 group was not enough to facilitate epimorphic cartilage formation. Minor elastic tissue damage can have a limited effect on the activation, migration, and proliferation of immune cells, fibroblasts, and smooth muscle cells. The formation of new elastic fibers may also depend on mechanical forces which help to align the fibers in the proper orientation. The damage stimulates the release of cytokines and growth factors that promote the migration and proliferation of cells to the site of injury. The presence of senescent cells and mechanical stress is necessary for complete epimorphic regeneration.

It is known that lectins are substances of plant origin that specifically bind to different cells and matrix carbohydrate residues [38,39]. We explored which lectins can be used for the staining of tissue structures in the newly formed cartilage. We used UEA I, WGA, SBA and RCA_120_ lectins, and found the characteristic binding of lectin RCA_120_ in the matrix of intact cartilage and in late-stage regeneration cells. Other lectins did not interact with the elastic cartilage structures of the rabbit due to the lack of a certain specificity. It is interesting that β-galactosidase hydrolyzes β-linked terminal galactosyl residues from a variety of substrates, including glycoproteins, glycolipids, and glycosaminoglycans. The hydrolysis product is β-D-galactose [40]. It is possible that β-D-galactose is used as an energy substrate for building new cells in regenerating cartilage. This hydrolysis product can be visualized by means of RCA_120_ lectin, which specifically binds to β-D-galactose [41,42]. We found that SA-β-galactosidase expression was inversely correlated with RCA_120_-positively stained cells. This can be explained by the fact that regeneration slows down after the partial elimination of senescent cells at the late stages, and β-D-galactose is not metabolized by cells in the same amount and accumulates. Moreover, β-D-galactose can be included in the composition of newly formed glycocalyx molecules during cartilage maturation, explaining the increase of RCA_120_ in chondrocytes at the later stages of regeneration. The production of β-D-galactose in the early period of regeneration was facilitated by high activity of β-D-galactosidase, but this metabolite was rapidly used as energy substrate for regeneration and was not detected by RCA_120_. However, the number of RCA_120_—positive cells can only indirectly indicate the rate and type of regeneration.

**Figure 12:**
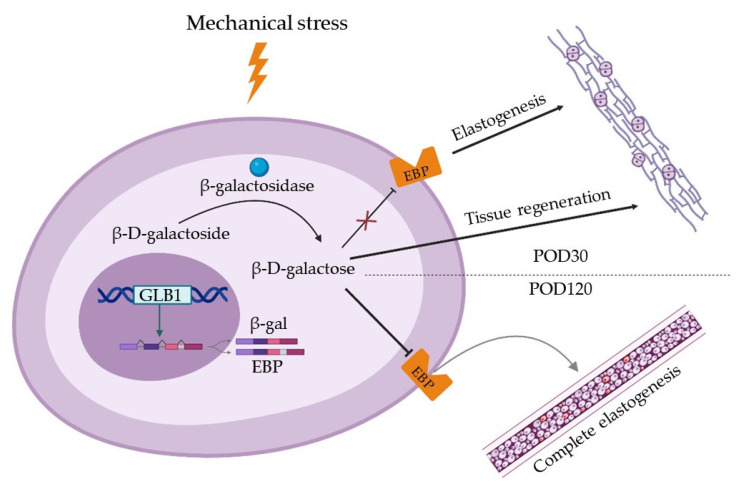
Mechanical stress regulates epimorphic regeneration of elastic cartilage through cellular senescence end production of elastic fibers. GLB1 gene encodes β-galactosidase and elastin binding protein (EBP). The course of translation is controlled by splicing. Mechanical stress drives gene translation into production of β-galactosidase and the increase of EBP formation stimulating proliferation of chondrocytes and growth of the newly formed tissue at early stages of regeneration. In the early stages, β-D-galactose is used as an energy substrate for building new cells in regenerating cartilage and does not inhibit EBP receptors. This allows for elastic fiber formation and cell proliferation. In the late stages, β-D-galactose metabolism slows down resulting in its accumulation and inhibition of EBP receptors.

It is important to note that elastic cartilage formed, replacing fibrous cartilage tissue over the course of regeneration. The number of elastic fibers increased linearly depending on time. Elastic fibers formed slowly in the newly formed cartilage over the first 60 days. Interestingly, β-galactosidase and the elastin binding protein (EBP), which is responsible for the elastin assembly, are products of alternative splicing of the GLB1 gene [40,43]. β-galactosidase, in turn, is an enzyme that isolates the terminal β-D-galactose from various compounds. β-D-galactose is able to bind specifically to the elastin binding protein [44]. This leads to the removal of the EBP from the cell surface, which eventually prevents elastic fiber formation. Elastin itself triggers signaling pathways responsible for activating cell proliferation and immune cell migration. Therefore, β-D-galactose is actively used to construct cartilage and less binds to the EBP in the early stages. As a result, elastin is actively synthesized and accumulates in cartilage in regeneration [44]. Our results indicate that β-D-galactose accumulates in cells and inhibits the formation of elastic fibers in the later stages of defect regeneration (Figure 12).

## 5. Conclusions

The regeneration of elastic cartilage requires a balance between the proliferative and synthetic activities of chondrocytes. Our results shed light on the senescent cells in regenerating tissue, and indicate the existence of therapeutic targets for accelerated and epimorphic regeneration. Further studies of the epimorphic regeneration of elastic cartilage will be focused on how cellular senescent cells can be affected by exogenous pro-regenerative factors. We envision the implementation of therapeutic approaches for the regulation of SASP cells in future tissue engineering materials and technologies for the reconstruction of anatomical structures in humans.

## Figures and Tables

**Table 1:** Score system for gross assessment of regeneration of cartilage defects.

Macroscopic Score	Wound Consolidation	Density	Wound Color	Surface Texture
0	Below 25%	Whole volume of the defect was dense	Red	Deformed with retractions and bulges
1	25–50%	Wound edges were dense	Pale	Smooth and even
2	50–75%	-	Pigmented	-
3	Above 75%	-	Normal	-

**Table 2:** Score system for evaluation of epimorphic regeneration of cartilage plate.

Epimorphic Regeneration Score	Morphological Features
0	Absence of round chondrocytes in regenerating fibrous cartilage
1	Presence of round chondrocytes in regenerating fibrous cartilage
2	Foci of regenerated elastic cartilage with large lacunae surrounded by elastic fibers
3	Continuous area of regenerated elastic cartilage with large lacunae surrounded by elastic fibers

**Table 3:** Score system for evaluation of elastic fiber content in regenerating cartilage tissue.

Elastic Fiber Regeneration Score	Morphological Features
0	Absence of elastic fibers
1	Separate foci (islands) of elastic fibers
2	Elastic fibers are located close to resident cartilage plate margins
3	Regenerating cartilage plate with elastic fibers

**Table 4:** Score system for evaluation of SA-β-galactosidase expression in regenerating cartilage tissue.

SA-β-Galactosidase Score	Morphological Intensity Features	Morphological Cell Quantity Features
**0**	Absence of SA-β-galactosidase positive cells	
**1**	Up to one positive granule in a cell	Singular SA-β-galactosidase positive cells
**2**	Several (up to four) positive granules in a cell	Foci of SA-β-galactosidase positive cells
**3**	Positive staining of the major part of the cell	Majority of SA-β-galactosidase positive cells

**Table 5:** Score system for evaluation of RCA_120_ expression in regenerating cartilage tissue.

RCA_120_ Score	Morphological Features
**0**	Absence of RCA_120_ positive cells
**1**	Up to one RCA_120_ positive cell in × 400 field of view
**2**	Two or more RCA_120_ positive cells located sparsely in × 400 field of view
**3**	Numerous RCA_120_ positive cells located in groups in × 400 field of view

## Data Availability

The relevant data generated and (or) analyzed in the current study is available from the corresponding author upon reasonable request.
